# Identification of the proliferative effect of Smad2 and 3 in the TGF β2/Smad signaling pathway using RNA interference in a glioma cell line

**DOI:** 10.3892/mmr.2015.3614

**Published:** 2015-04-15

**Authors:** CHENGYUAN DONG, RUIFANG MI, GUISHAN JIN, YIQIANG ZHOU, JIN ZHANG, FUSHENG LIU

**Affiliations:** Brain Tumor Research Center, Beijing Neurosurgical Institute and Department of Neurosurgery, Beijing Tian Tan Hospital, Capital Medical University, Beijing 100050, P.R. China

**Keywords:** glioma, transforming growth factor β2, small mothers against decapentaplegic 2, small mothers against decapentaplegic 3, transfection, proliferation

## Abstract

Gliomas are the most frequently occurring primary tumor in the brain. The most malignant form of glioma, glioblastoma multiforme (GBM), is characterized by rapid and invasive growth and is restricted to the central nervous system (CNS). The transforming growth factor β2 (TGFβ2)/small mothers against decapentaplegic (Smad) signaling pathway is important, not only in GBM cell metabolism and invasion, but also in GBM cell proliferation. However, the functions of the downstream mediators of the TGFβ2/Smads signaling pathway remain to be fully elucidated. In the present study, short hairpin (sh)RNA interference was used to specifically inhibit the expression of Smad2 and Smad3 in the TGFβ2/Smad signaling pathway to investigate the effects of shRNA on the proliferation of human GBM cells. The results demonstrated that knockdown of either Smad2 or Smad3 enhanced cellular proliferation. Additionally, the key target genes involved in GBM cell proliferation, induced by TGFβ2, were found to be dependent on Smad3, but not Smad2.

## Introduction

Gliomas are the most common type of primary tumor of the brain. They are classified into four clinical grades, according to histology and prognosis, by the World Health Organization (1). The most malignant form, Grade IV glioma, is glioblastoma multiforme (GBM). The survival rate of patients with GBM, has not improved since the 1980s, with a 1-year relative survival rate of ~30% and a 5-year survival rate of <5% (2). Even with comprehensive treatment, which includes surgery, chemotherapy and irradiation, the prognosis and treatment of GBMs remains limited (3).

Among several inflammatory cytokines, the transforming growth factor (TGF)β family has been implicated in glioma. There are five subclasses of the TGFβ family: β1, β2, β3, β4 and β5. TGFβ1, β2 and β3 are expressed in mammalian tissues (4). Within the TGFβ family, TGFβ2 is the most potent factor, which is involved in the initiation and maintenance of GBM (5).

TGFβ family cytokines affect a diverse array of cellular processes, including cellular proliferation, differentiation, apoptosis and migration (6). Once activated, TGFβ binds and activates the TGFβ type II receptor, TβRII, and the TGFβ type I receptor, TβRI, which phosphorylates Smad2 and Smad3 to generate phosphorylated (p-)Smad2 and p-Smad3. Upon phosphorylation, Smad2 and Smad3 form transcriptional complexes with Smad4 and other transcription factors and accumulate in the nucleus, where they regulate transcription (7,8).

The Smad family of proteins can be divided into three different subfamilies: The receptor-activated Smads (R-Smads), common mediator Smads and inhibitory Smads (9). Smad2 and Smad3 are R-Smads and are directly phosphorylated in response to TGFβ and activin (10). Although Smad2 and Smad3 belong to the R-Smad subfamily and are important in the biological effects of TGFβ, they do not have identical effects in cellular proliferation.

A previous study, involving a human lens cell line, revealed that the effect of TGFβ2 on cell proliferation depends on the Smad3 signaling pathway (11). Another study found that Smad2 and Smad3 have different roles in pancreatic ductal adenocarcinoma cells (12).

As mentioned above, Smad2 and Smad3 have different functions, however, the specific functions of Smad2 and Smad3 in GBM remain to be elucidated, as does whether each response is mediated predominantly or exclusively by only one of the two R-Smads. The predominant focus of the present study was to determine the specific roles of Smad2 and Smad3 in the proliferative effect of TGFβ in GBM.

Therefore, the present study investigated how downregulation in the expression levels of Smad2 and Smad3 affected glioma cell proliferation, and whether GBM cell proliferation was controlled differentially by Smad2 and Smad3. For this purpose, the well-characterized U251 GBM cell line, which has retained a functional TGFβ2/Smad pathway, was used (13). RNA interference was used to specifically knock down the expression of the two R-Smads to determine whether TGFβ2 proliferation was dependent on the Smad3 and/or Smad2 signaling pathway.

## Materials and methods

### Cell lines and cell culture

U251 cells were purchased from American Type Culture Collection (Manassas, VA, USA). The U251 cells were cultured in Dulbecco’s modified Eagle’s medium (DMEM; Gibco-BRL, Grand Island, NY, USA), supplemented with 10% fetal bovine serum (FBS; Gibco-BRL) at 37°C in a humidified 5% CO_2_ atmosphere.

### Short hairpin (sh)RNA transfection

The cells were grown to 70–90% confluence, at the time of transfection. Smad2 and Smad3 shRNAs were synthesized and inserted into the pGPU6/GFP/Neo empty vector plasmid to construct the pGPU6/GFP/Neo-Smad2-shRNA (psh-Smad2) and pGPU6/GFP/Neo-Smad3-shRNA (psh-Smad3) plasmids by Shanghai GenePharma (Shanghai, China). The pGPU6/GFP/Neo-negative control-shRNA (psh-NC) non-silencing control plasmid was also synthesized by GenePharma and was confirmed by BLAST analysis (http://blast.ncbi.nlm.nih.gov.ezp.lib.unimelb.edu.au/Blast.cgi) to have no complementarity to any mammalian mRNA sequence. The following gene-specific sequences were used: 5′-GCAGAACTATCTCCTACTACT-3′ for Smad2, 5′-GGCTGCTCTCCAATGTCAACA-3′ for Smad3 and 5′-GTTCTCCGAACGTGTCACGT-3′ for NC shRNA. The gene-specific sequences were generated by Shanghai GenePharma. The plasmids were transfected using Lipofectamine^®^ 2000 (Invitrogen Life Technologies, Carlsbad, CA, USA), according to the manufacturer’s instructions. Transfection efficiency was determined using an inverted microscope (DMI3000B; Leica Microsystems, Wetzlar, Germany).

### Total RNA extraction, cDNA synthesis and reverse transcription-quantitative polymerase chain reaction (RT-qPCR)

The expression levels of Smad2 and Smad3 were determined by qPCR. The cells were collected 24 h post-transfection (80–90% confluence). The total RNA from the cultured tumor cells was isolated using TRIzol reagent, according to the manufacturer’s protocol (Invitrogen Life Technologies). RT was performed to generate cDNA using the PrimeScript^®^ RT-PCR kit (Takara Bio, Inc., Otsu, Japan).

For qPCR, SYBR^®^ Premix Ex Taq™ II (Takara Bio, Inc.) was used, according to the manufacturer’s instructions, using a 7500 Fast Real-Time PCR system (Applied Biosystems, Foster City, CA, USA). Each 20 *μ*l reaction contained ~100 ng DNA template and 0.4 *μ*M of the forward and reverse primers (Invitrogen Life Technologies). The GAPDH gene served as an endogenous reference. The primer sequences used in the present study are listed in Table I.

The thermocycler parameters were as follows: 95°C for 30 sec, followed by 40 cycles of 95°C for 3 sec and 56°C for 30 sec. The qPCR reactions were performed in triplicate and the relative mRNA expression levels were quantified based on the threshold cycle (Ct) value, normalized to GAPDH, and expressed as relative quantities. The quantity of the target normalized to GAPDH and relative to the calibrator target gene in the TGFβ2 group, was calculated using the following formula: Fold-change in TGFβ2 (+/−) = 2^−ΔΔCT^, where ΔΔC_T_ = ΔC_TT_ − ΔC_TC_ (ΔC_T_ = C_Ttarget_ − C_Treference_) (14). Where ΔC_TT_ represents the ΔCt value of the target, and ΔC_TC_ represents the ΔCt value of the control.

### Western blot analysis

At 24 h post-transfection, protein was extracted from the cultured cells using radioimmunoprecipitation assay lysis buffer containing 1% protease inhibitors (Roche Diagnostics, Basel, Switzerland), the cellular proteins were then boiled at 100°C for 10 min. The protein concentration was determined using Thermo Scientific NanoDrop 2000 (Thermo Scientific, Waltham, MA, USA). Equal quantities of protein (50 ng) were subjected to a 10% SDS-PAGE (Applygen Technologies, Inc., Beijing, China) and then transferred to a nitrocellulose membrane (Millipore, Bedford, MA, USA). The membranes were blocked with 5% skim milk (Applygen Technologies, Inc.) for 2 h at room temperature and incubated with rabbit anti-human monoclonal Smad2 (cat. no. 3122) and Smad3 (cat. no. 9523) antibodies (1:1,000; Cell Signalling Technology, Inc., Heidelberg, Germany) or GAPDH antibody (1:1,000; cat. no. 2118; Cell Signalling Technology, Inc.) in 5% skim milk overnight at 4°C. Following incubation, the membranes were washed three times in phosphate-buffered saline (PBS) containing 0.1% Tween-20 (PBST) for 10 min. The membranes were then incubated with a goat anti-rabbit antibody (1:1,000; cat. no. ZDR-5306; Applygen Technologies, Inc.) for 90 min at room temperature. Following three further washes in PBST, the protein expression levels were determined by enhanced chemiluminescence (Applygen Technologies, Inc.) and exposure to chemiluminescent film (Applygen Technologies, Inc.).

### Cell proliferation assay

At 24 h post-transfection, a Cell Counting kit-8 (CCK-8; Dojindo Molecular Technologies, Kunamoto, Japan) was used to determine the effect of TGFβ2 (R&D Systems, Inc., Wiesbaden, Germany) on the proliferation of the transfected cells. The cells (3×10^3^) cells were seeded in 96-well plates and cultured in DMEM without FBS at 37°C for 24 h. The culture medium was then removed. Subsequently, the cells were treated with or without 1.3 ng/ml TGFβ2 in DMEM for 12 h at 37°C. Subsequently, 10 *μ*l CCK-8 dye was add to each well and the cells were incubated at 37°C for 1 h. The absorbance at 450 nm was determined using a multimode reader (Wellscan MK3; Labsystems Dragon, Helsinki, Finland). Three parallel experiments for each sample were used to assess cell proliferation.

### Statistical analysis

Statistical analyses were performed using the SPSS software package (version 16.0; SPSS, Inc., Chicago, IL, USA). The results were analyzed using one-way analysis of variance and a least significant difference t-test at a global level of significance of 95%. The data are presented as the mean ± standard deviation. P<0.05 was considered to indicate a statistically significant difference.

## Results

### Specific silencing of the TGFβ2/Smad2 or TGFβ2/Smad3 signaling pathway using shRNA

**Figure 1:**
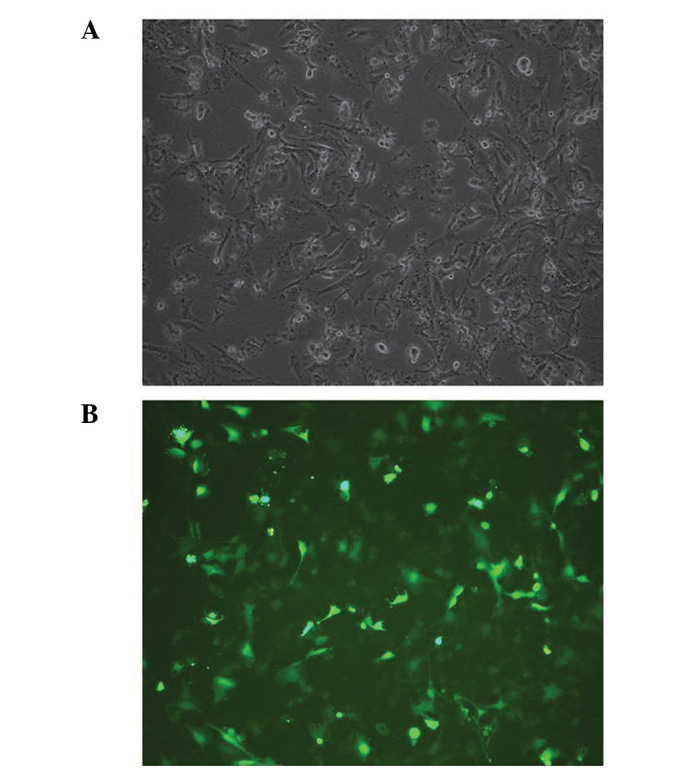
Expression of GFP in U251 cells transfected with shRNAs carrying the pGPU6/GFP/Neo plasmid (magnification, ×200). (A) Optical microscopy. (B) Fluorescence microscopy revealing cells exhibiting GFP emission (magnification, ×100). shRBA, short interference RNA; GFP, green fluorescent protein.

**Figure 2:**
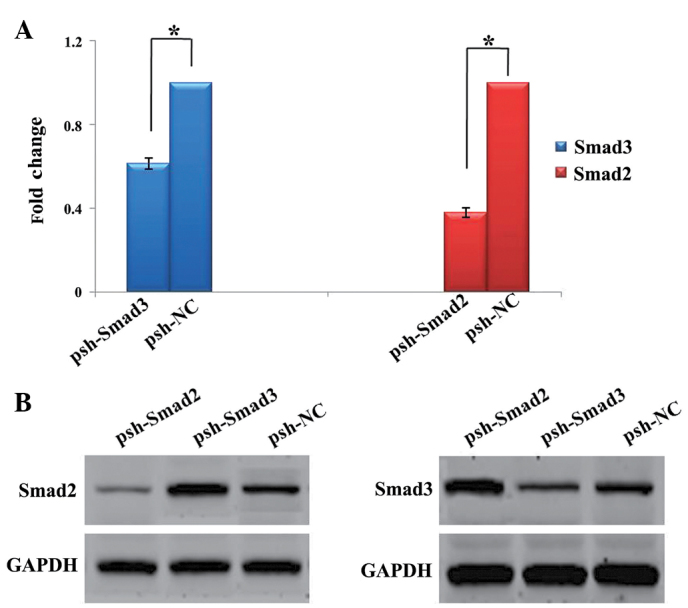
Inhibitory effect of shRNAs, determined by (A) RT-qPCR and (B) western blot analysis in U251 cells. (A) Representative images of RT-PCR analysis of the mRNA expression levels of Smad2 and Smad3 in the U251 cells. The mRNA levels of Smad2 and Smad3 were significantly decreased by psh-Smad2 and psh-Smad3 (^*^P=0.017 and ^*^P=0.002, respectively). The data are presented as the mean ± standard deviation. (B) Representative images of the western blot analysis of the protein expression levels of Smad2 and Smad3 in the U251 cells. The protein expression levels of Smad2 and Smad3 in the U251 cells were inhibited by psh-Smad2 and psh-Smad3. GAPDH was used as an internal loading control. RT-qPCR, reverse transcription-quantitative polymerase chain reaction; shRNA, short hairpin RNA; Smad, small mothers against decapentaplegic; NC, negative control.

To confirm the effect of the constructed shRNAs, the shRNA expression vectors were transfected into U251 cells. The transfection efficiency was confirmed using an inverted microscope, and the results demonstrated that the pGPU6/GFP/Neo vector, which contained a cassette of GFP, had been successfully transfected into the U251 cells (Fig. 1). The expression levels of Smad2 and Smad3 were detected using an RT-qPCR assay and western blot analysis. The psh-NC plasmid was used as a control. The results of the qPCR and western blot analyses revealed that Smad2 and Smad3 were specifically and efficiently knocked down by their corresponding shRNA (Fig. 2).

### Depletion of Smad2/3 enhances cell proliferation

**Figure 3:**
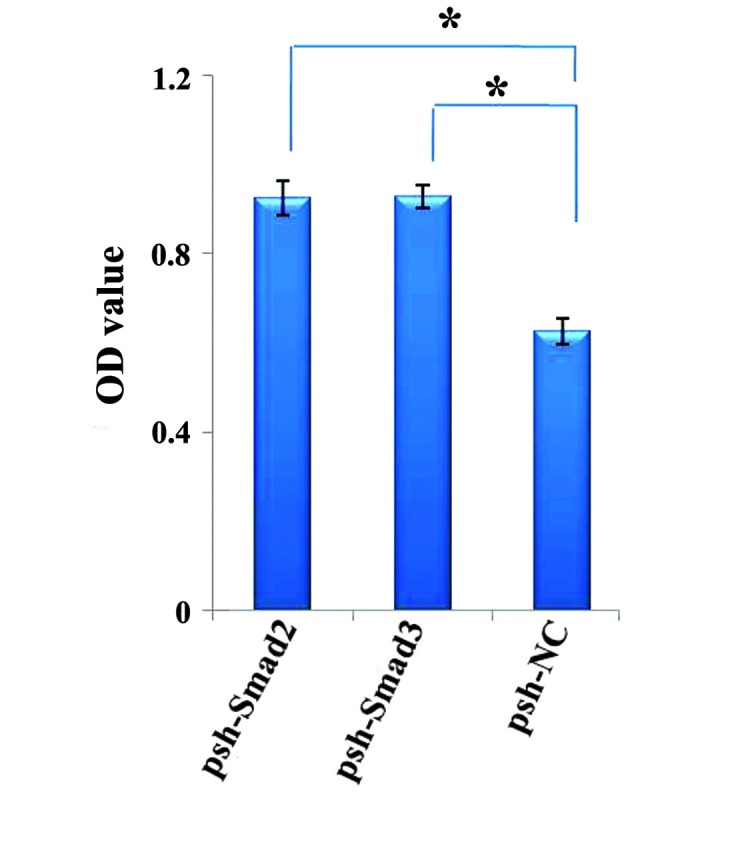
Cell proliferation assay for the U251 cells treated with shRNAs. (A) U251 cells were transfected with Smad2 shRNA (psh-Smad2), Smad3 shRNA (psh-Smad3) or negative control shRNA (psh-NC). Cell proliferation was significantly increased in the cells transfected with either psh-Smad2 or psh-Smad3, compared with those transfected with psh-NC. OD, optical density; shRNA, short hairpin RNA; NC, negative control.

The present study investigated how downregulation of the expression levels of Smad2 and Smad3 affected glioma cell proliferation. Therefore, following transfection of the cells with Smad2 shRNA or Smad3 shRNA, the cell growth response to 24 h treatment with DMEM was determined using a CCK-8 assay. A significant increase in absorbency at 450 nm was observed in the U251 cells transfected with Smad2 shRNA (P<0.000) or Smad3 shRNA (P<0.000; Fig. 3). This result suggested that silencing Smad2 or Smad3 may promote cell proliferation.

### Induction of cell proliferation by TGFβ2 depends on the TGFβ2/Smad3 signaling pathway

**Figure 4:**
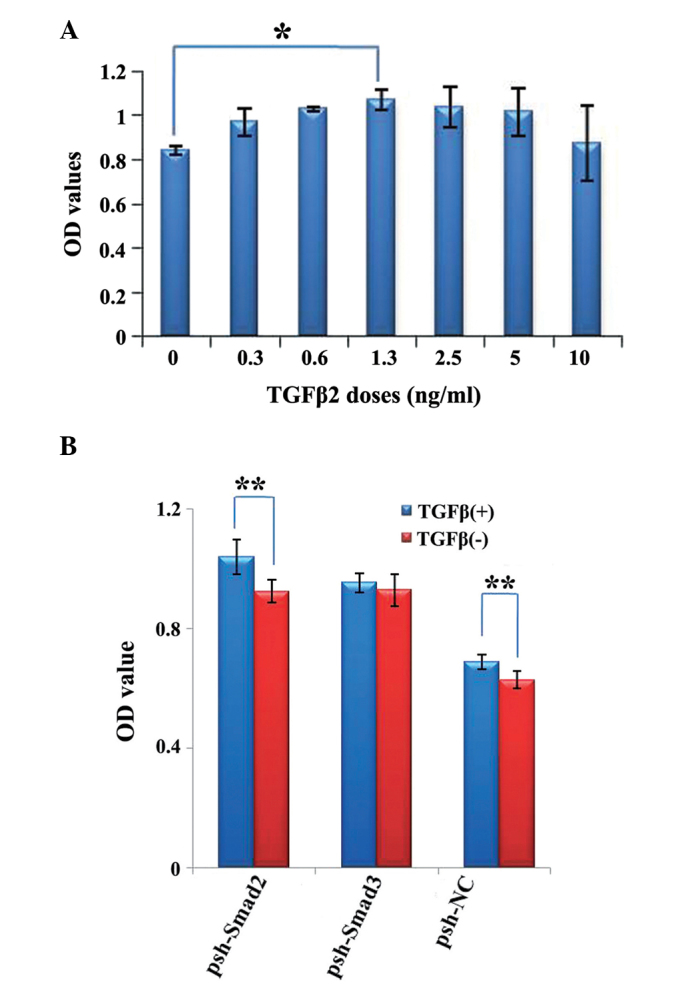
Cell proliferation assay for U251 cells transfected with shRNAs +/− TGFβ2. (A) To determine the optimal concentration of TGFβ2 in the U251 cells. The cells were treated with increasing doses of TGFβ2. The optimal concentration was determined as 1.3 ng/ml, at which the OD was highest (^*^P=0.008, compared with the untreated control. (B) Following transfection with psh-Smad2, psh-Smad3 or psh-NC, the cells were treated with (+) or without (−) TGFβ2 and the cell proliferation activity was detected. The proliferation of cells transfected with psh-Smad2 or psh-NC differed significantly in the presence of TGFβ2 (P=0.001 and P=0.009, compared with TFFβ2−). The proliferation of the cells transfected with psh-Smad3 was similar in the presence or absence of TGFβ2 (P=0.258). Data are presented as the mean ± standard deviation. OD, optical density; TGF, transforming growth factor; shRNA, short hairpin RNA; NC, negative control.

To determine the optimal concentration of TGFβ2 in the present study, the U251 cells were treated with increasing doses of TGFβ2 for 24 h and rapid TGFβ2 responses, which were more likely to be direct, were determined to identify the optimal concentration of 1.3 ng/ml (Fig. 4A).

It is widely accepted that the TGFβ2/Smads signaling pathway is involved in the regulation of cell proliferation (15). However, the relative involvement of Smad2 and Smad3 in the control of TGFβ2-induced cell proliferation in glioma cells remains to be elucidated. Therefore, the present study investigated the mechanism underlying the induction of proliferation by TGFβ2/Smads in glioma. The U251 cells were transfected with Smad2 shRNA, Smad3 shRNA or NC shRNA, and the growth response following 12 h treatment with or without TGFβ2 was assessed using a CCK-8 assay. The proliferation rates of the cells transfected with Smad2 shRNA or NC shRNA increased in the presence of TGFβ2 (P=0.001 and P=0.009, respectively). However, the rate of cell proliferation was similar between the cells treated with or without TGFβ2 when the Smad3 signaling pathway was inhibited (P=0.258; Fig. 4B). These results demonstrated that the promoting effect of TGFβ2 on cell proliferation was dependent on the Smad3 signaling pathway.

## Discussion

Glioma is the most common type of primary intracranial malignancy, and GBM is the most malignant type of gliom, accounting for ~70% of malignant brain tumors in adults (16). Despite advances in treatment, including surgical resection followed by concurrent chemotherapy with radiation, GBM remains an incurable and life-threatening disease, with a median survival rate of ~9–15 months following diagnosis (17). TGFβ proteins regulate cell function and are important in development and carcinogenesis (18). Although the downstream signaling events, which occur to stimulate cell proliferation remain to be fully elucidated, the intracellular effectors of TGFβ signaling, the Smad proteins, are activated by receptors and are translocated into the nucleus, where they regulate transcription (19).

Previous studies have investigated the expression levels of Smad2 and Smad3 in gliomas in tumor specimens and cell lines, however, the results have been inconsistent. Zhang *et al* examined the expression levels of downstream components of the TGFβ receptor, including Smad2 and Smad3, in 10 glioma cell lines. The results revealed that the protein expression levels of Smad2 and Smad3 were lower in the glioma cell lines compared with normal astrocytes (20). Similarly, Kjellman *et a*l analyzed the mRNA expression levels of Smad2 and Smad3 in tissue specimens from 23 cases of glioma, in which decreased mRNA levels of Smad2 and Smad3 were observed and correlated with the degree of malignancy (5). However, a study by Horst H *et al* found that the mRNA expression levels of Smad2 and Smad3 increased with the degree of malignancy (21).

To examine the effects of the downstream components of the TGFβ2/Smads signaling pathway, Smad2 and Smad3, on GBM cell proliferation, the present study transfected U251 cells with shRNAs, to selectively deplete Smad2 and Smad3, and measured the growth of the cells. The results revealed that the knock down of Smad2 and Smad3 enhanced cellular proliferation, demonstrating that Smad2 and Smad3 had an inhibitory effect on cell proliferation in this glioma cell line. A study by Zhang *et al* demonstrated that the protein expression levels of Smad2 and Smad3 were lower in glioma cell lines compared with normal astrocytes (20). Considering these results, the preset study hypothesized that the ability to resist TGFβ2-mediated growth inhibition in malignant glioma cells was due to a decrease in the expression levels of Smad2 and Smad3 in the TGFβ2 signaling pathway.

There is now substantial evidence that Smad2 and Smad3 have distinct functions in TGFβ signaling. Inhibiting the function of endogenous Smad3 in ductal adenocarcinoma, liver and human lens cell lines significantly suppresses the effect of TGFβ on cell proliferation (11,12,20). However, there is no evidence that Smad2 and Smad3 have distinct functions in GBM growth.

The present study aimed to confirm the functions of Smad2 and Smad3 in GBM cells by transfecting U251 cells with shRNAs to selectively deplete Smad2 and Smad3, and analyzing the proliferative response of the cells to TGFβ2 using a CCK-8 assay. The results revealed a difference in the rate of cell proliferation between the cells with and without TGFβ2 following transfection with Smad2 shRNA or NC shRNA. However, the rate of cell proliferation was similar between the cells treated with and without TGFβ2 when the Smad3 signaling pathways were inhibited. These results demonstrated that Smad3 was more important in the regulation of TGFβ2-inducedf cell proliferation in glioma cells. Previous studies have reported that Smad2 contains an extra exon in the MH1 domain, absent from Smad3, which encodes 30 amino acids and interferes with DNA recognition; thus, Smad3 can interact directly with Smad-binding element sequences in DNA (22). Based on these studies and the results of the present study, we hypothesized that the differences in the molecular structures of Smad2 and Smad3 may be the reason underlying why Smad3 has a significant effect on the regulation of TGFβ2-induced cell proliferation in glioma cells. However, further investigations experiments are required to confirm these findings.

In conclusion, the present study provided evidence that the Smad3 pathway is important in malignant glioma cells and suggested that Smad2 and Smad3 have tumor suppressor activities. Therefore, the proliferation of GBM cannot be prevented by inhibiting the TGFβ2/Smad2 and 3 signaling pathway. Although further studies are required, these results may provide a reference in attempts to modulate the growth of malignant gliomas.

## Figures and Tables

**Table I:** Primers used for reverse trasnscription-quantitative polymerase chain reaction.

Primer	Sequence	Product length (bp)
Smad2	F: 5′-ACTAACTTCCCAGCAGGAAT-3′ R: 5′-GTTGGTCACTTGTTTCTCCA-3′	40
Smad3	F: 5′-CCACGCAGAACGTCAACA-3′ R: 5′-TTGAAGGCGAACTCACACAG-3′	38
GAPDH	F: 5′-GAGTCAACGGATTTGGTCGT-3′ R: 5′-TTGATTTTGGAGGGATCTCG-3′	40

F, forward; R, reverse. Smad, small mothers of decapentaplegic.
